# Monocytes in Uremia

**DOI:** 10.3390/toxins12050340

**Published:** 2020-05-21

**Authors:** Matthias Girndt, Bogusz Trojanowicz, Christof Ulrich

**Affiliations:** Department of Internal Medicine II, Martin-Luther-University Halle-Wittenberg, 06120 Halle, Germany; bogusz.trojanowicz@uk-halle.de (B.T.); christof.ulrich@uk-halle.de (C.U.)

**Keywords:** chronic kidney disease, cytokines, hemodialysis, inflammation, monocytes, uremic toxins

## Abstract

Monocytes play an important role in both innate immunity and antigen presentation for specific cellular immune defense. In patients with chronic renal failure, as well as those treated with maintenance hemodialysis, these cells are largely dysregulated. There is a large body of literature on monocyte alterations in such patients. However, most of the publications report on small series, there is a vast spectrum of different methods and the heterogeneity of the data prevents any meta-analytic approach. Thus, a narrative review was performed to describe the current knowledge. Monocytes from patients with chronic renal failure differ from those of healthy individuals in the pattern of surface molecule expression, cytokine and mediator production, and function. If these findings can be summarized at all, they might be subsumed as showing chronic inflammation in resting cells together with limited activation upon immunologic challenge. The picture is complicated by the fact that monocytes fall into morphologically and functionally different populations and population shifts interact heavily with dysregulation of the individual cells. Severe complications of chronic renal failure such as impaired immune defense, inflammation, and atherosclerosis can be related to several aspects of monocyte dysfunction. Therefore, this review aims to provide an overview about the impairment and activation of monocytes by uremia and the resulting clinical consequences for renal failure patients.

## 1. Introduction

Monocytes are bone marrow derived cells that circulate in the blood for 1–3 days before differentiating into tissue macrophages or dendritic cells. They are part of the innate immune system and react quickly but unspecifically to any tissue damage or pathogen intrusion. Monocytes can act as phagocytes that take up pathogens. They produce cytokines that regulate inflammatory responses. Among their most important functions are chemotaxis, which describes the approach to sites of tissue damage in response to soluble mediators and cytokines, as well as adhesion and transmigration through the endothelium. The latter is promoted by specific adhesion molecules that bind to endothelial ligands. Monocytes also play an important role as antigen-presenting cells. They can present foreign antigens towards lymphocytes thereby activating the adaptive immune response. In addition, they provide secondary signals to the lymphocytes to regulate the immune reaction. Such secondary signaling can adjust a response towards an antigen from tolerance to cytolytic attack. Monocytes are precursors of dendritic cells, the professional and highly efficient antigen-presenting cells of the immune system.

Monocytes fall into three different subgroups that can be distinguished via the expression pattern of the receptor for lipopolysaccharide (CD14) and the immunoglobulin fc segment receptor CD16 [1]. These populations differ in function and cytokine spectrum [2]. The populations are termed Mo1 (“classical monocytes” expressing high levels of CD14 only), Mo2 (“intermediate monocytes”, CD14++/CD16+), and Mo3 (“nonclassical monocytes, CD14+/CD16++). The functional and morphological heterogeneity of these populations needs to be considered when describing surface marker expression as well as cytokine production by monocytes from patients with chronic renal failure. Differences between healthy individuals and CKD patients may be caused by dysregulation of the cells or by alterations in the relative number of the three different populations in the blood. Unfortunately, this aspect was considered only by a minority of publications in this field.

In the recent 25 years there have been many publications focusing on abnormal structure or function of monocytes in patients with CKD. These studies were done to elucidate the diminished immune defense function that lead to frequent infections in these patients. They also address the causes of chronic inflammation, which is found in the majority of these patients, even in the absence of infection. This review aims firstly to describe the structural and functional abnormalities observed in monocytes from CKD patients, secondly to review the mechanism by which CKD affects monocytes populations and fundamental monocytic functions and thirdly to explain how these alterations may impact both antimicrobial defense and atherosclerosis development in this population.

## 2. Phenotype of Monocytes in CKD

Most studies describe normal numbers of monocytes in the circulation, while there was a tendency towards lymphopenia [3,4,5]. While the cells are quantitatively available in the blood, there are numerous alterations in their phenotype and function that contribute to immune dysfunction in CKD. Further, the alterations in monocyte function may also be relevant to the pathogenesis of enhanced cardiovascular disease in these patients. In general, uremia shifts monocytes to a CD16-positive phenotype, whereby these cells are smaller in size, inflammatory, adhesive, and senescent [6,7,8].

### 2.1. Surface Marker Expression

Several studies over the years attempted to characterize monocytes in renal failure patients by detecting the surface expression pattern of typical receptors or membrane bound proteins. The intention was to detect differences in comparison with cells from healthy individuals to draw conclusions on potential functional disruptions. Part of the relevant literature dates back to between 1995 and 2005. Only after that time it became known that monocytes fall into distinct populations and that the quantitative distribution of these populations is affected by CKD. Thus, in the older studies, effects of population distribution cannot be distinguished from alterations of surface marker expression per cell.

Table 1 lists the findings on surface marker expression in patients on hemodialysis in comparison with healthy individuals. It is difficult to draw firm conclusions from these data. First, the studies were done over several decades with changing dialysis technology and treatment modalities. In addition, many of the studies were quite small. With these limitations in mind, a conclusion from the surface marker analysis might be that monocytes show signs of activation, are more prone to react to inflammatory cytokines (e.g., TNF) and oxidized lipoproteins (CD36), and have enhanced ability to adhere and potentially transmigrate the endothelium (CD11b). The enhanced migration capacity of monocytes is suggested by increased chemokine receptor expression of CCR2 and CX3CR1. High expression of ACE may be considered as a further requisite of these cells to produce inflammatory mediators. Enhanced expression of CD40 might be important for cardiovascular diseases.

### 2.2. Cytokine and Mediator Secretion

Several studies compared the secretion of cytokines and other mediators by monocytes from patients with CKD with those from healthy individuals. In most cases, these studies purified monocytes by different methods from blood samples, took them into cell culture, and measured the secretion of these mediators into the cell culture supernatant. Table 2 lists the results of these studies.

A number of earlier studies also described enhanced stimulated secretion of monocyte-derived cytokines, e.g., TNF-α [33], IL-1ß [34,35], IL-1-RA [34], IL-6, and soluble IL-6 receptor [36], TNF-α, IL-8, and the regulatory cytokine IL-10 [37,38]. However, these studies analyzed supernatants from unfractionated leukocytes which makes it difficult to assess the contribution of the monocytes to these findings. These studies will not be considered here in more detail.

Although the literature is ambiguous, it seems safe to conclude that resting monocytes in the circulation of patients with CKD have a higher level of activation than in healthy individuals. Several proinflammatory cytokines are spontaneously secreted into culture supernatant indicating that these cells might contribute to chronic inflammation in the patients. When stimulated by endotoxin (lipopolysaccharide, LPS), these cells cannot enhance cytokine production in the same way as cells from healthy donors do. This might be interpreted as a state of exhaustion.

Again, as with the studies on surface marker expression, monocyte population shifts might contribute to these findings. Studies measuring cytokines in cell culture supernatants of a defined number of monocytes assume that all cells contribute equally. However, later studies showed that this assumption is not true [28,31]. It has been shown by flow-cytometry that the number of cytokine producing monocytes increases while the amount produced by each cell remained unchanged [28].

### 2.3. Monocyte Function and Expression of Functional Proteins

Monocytes not only secrete cytokines, they also provide several functional proteins that are needed during inflammation to regulate cellular homeostasis. Among these proteins are proinflammatory (ACE, Lp-PLA2) as well as antioxidative substances (SOD1, SOD2). Further cellular systems such as the angiotensin system (ACE, AT1-R, AT2-R) balance functional activation. Table 3 shows the results of studies addressing the expression of functional proteins, most of them measuring transcriptional activation.

CKD and particularly hemodialysis treatment are associated with oxidative stress [6]. The functional alterations regarding antioxidative systems reflect this chronic activation. Such findings can be interpreted as the chronic exhaustion of the protective and regulatory systems with mainly reduced protein content as well as impaired transcription. Interestingly, for several parameters this impairment of protective systems is stronger in dialysis patients than in those with different stages of CKD [39,42]. In contrast, the SOD2 system seems to recover once the patient is on renal replacement therapy [40]. On the other hand, production of reactive oxygen species (ROS) by monocytes is an important mechanism of antibacterial defense by which granulocytes and monocytes respond to Staph. aureus or fungal infections. CKD and dialysis patients are at enhanced risk of staphylococcus sepsis. The studies showed that both ROS production in response to Staphylococcus aureus [38] and phagocytic capacity are impaired in dialysis patients [46]. Thus, the inability of monocytes to mount an adequate response might contribute causally to the high sepsis risk in renal failure patients. With these data, one should keep in mind that monocytes can participate in ROS formation and phagocytosis, however, they are not the “professional” cell type that is quantitatively important for these aspects of antibacterial defense.

Monocytes express several components of the angiotensin system [47], which is mostly known in conjunction with blood pressure and electrolyte regulation. In CKD, monocytes express high levels of the ACE enzyme that converts angiotensin I to angiotensin II. This might be a link between cardiovascular disease and monocyte activation in patients with CKD [25]. However, not only ACE is overexpressed, the entire system of angiotensin modifying enzymes and angiotensin II receptors shows a dysregulated pattern [48]. Treatment with the angiotensin receptor blocker Losartan attenuated monocyte activation in hemodialysis patients [49]. These findings constitute a promising new research area at the boundary between inflammation and atherosclerosis, particularly since the angiotensin system is therapeutically accessible.

Monocytes from patients with CKD and on dialysis treatment show higher apoptosis rates per time than monocytes from healthy controls [50]. This programmed cell death contributes to loss of phagocytic function over time.

## 3. Monocyte Subpopulations

**Figure 1:**
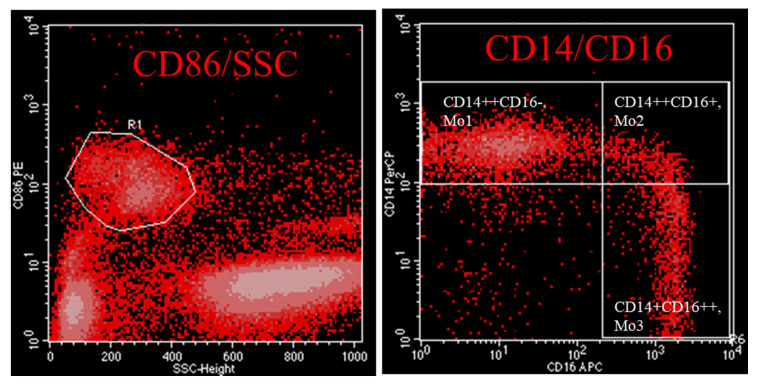
Flow cytometry panel showing gating strategy of monocytes of an uremic patient by anti-CD86 staining and subdivision of monocytes in classical (Mo1), intermediate (Mo2) and non-classical Mo3 according anti-CD14/CD16 staining. R1 includes CD86+ monocytes.

Monocytes are not a homogenous cell type in the circulation. They fall into three distinct populations that have different functions and can be distinguished upon their surface molecule expression pattern. The phenotypic characterization of monocytes can easily be performed by flow-cytometry. Monocytes are staining negative for anti-CD3, -CD19, -CD20, -CD56 and -CD66. According to Ziegler-Heitbrock et al. monocytes are staining positive for HLA-DR and can be subdivided into “classical” (Mo1), “intermediate”, Mo2, or “non-classical”, (Mo3) [1]. An alternative strategy to characterize monocyte subsets favours staining of cells with the pan-monocyte marker CD86 in combination with CD14 and CD16 (Figure 1, [25]). Analysis of gene expression patterns show that Mo2 differ from Mo1 and Mo3 in three predominant aspects: as they express genes encoding MHC class II molecules such as HLA-DR and CD74, Mo2 are predisposed to antigen expression. Further on, Mo2 have a high inflammatory capacity as they express genes such as TGFB1, AIF1 and PTPN6. Finally, since they express the markers Tie2 and CD105, they seem to be involved in angiogenesis [2]. Because of these reasons, it seems justified to label them “inflammatory monocytes”.

Nockher and Scherberich [51] were the first to point out that there is an expansion of Mo2 cells among the monocytes of patients with CKD. This population accounts for some 8% of the monocytes in healthy individuals while it is expanded up to 18% in CKD. Expansion of the Mo2 population was confirmed by several other groups [31,52,53,54,55,56,57]. The presence of diabetes mellitus seems to accentuate the population expansion in patients with CKD [58].

The expansion of the Mo2 population is associated with cardiovascular disease in dialysis patients [52]. This might be related to the fact that Mo2 cells have particular proinflammatory activity, and systemic inflammation is associated with cardiovascular disease in general. A further interesting finding is that especially CD16+ (Mo2 and Mo3) cells are more prone to attach to endothelial cells in coculture experiments [59]. These cells also appear to be associated with endothelial damage in vivo as Merino et al. found an elevated frequency of CD 16+ monocytes together with increased levels of CD31+Annexin+ microparticles (EMP) in the blood of CKD patients. EMP are considered as an early marker of endothelial damage [60].

The analysis of monocyte populations has revolutionized research on this cell type in CKD. It became obvious that all previous findings on surface marker expression and cytokine secretion need to be reconsidered since they were made with the assumption that monocytes are a homogenous cell type. While up- or downregulation of a marker may be the result of increased or decreased expression per cell, it may well be that this finding merely reflects alterations in monocyte population distribution. To date, several studies have shown clearly that Mo2 cells differ from Mo1 and Mo3 in surface marker expression and function. This was particularly demonstrated for ACE [61], or TLR-9 [62] (both highly expressed on Mo2).

Some studies addressed the question if expansion of the CD14^++^CD16^+^ cells can be induced by incubating PBMC in vitro with plasma from patients with CKD [63]. This question cannot be answered yet even though the cited study claimed to see expansion in this setting. Most likely, population shifts occur in the organism by sequestration of cells and release from bone marrow and not by differentiation later on [64]. Nevertheless, uremic toxins may also play a role. At least patients with preserved residual renal function seem to have less expansion of Mo2 cells [65]. Studies on different dialysis modalities found conflicting results. While online-hemodiafiltration (HDF) was reported to reduce the Mo2 population [66,67,68], this could not be demonstrated for high cut-off hemodialysis [69] in prospective trials. In addition to differences in clearance of retention solutes, biophysical aspects of the treatment might be relevant as well. This was suggested by a recent trial comparing mixed vs. postdilution HDF [66]. Potentially, the different hemoconcentration and shear stress contributed to the monocyte population distribution.

## 4. Causes and Pathogenesis of Monocyte Alterations

Since many of the morphological and functional alterations of monocytes are found in both CKD 3–5 and dialysis patients, uremic toxicity may be one of the important causal factors. However, hemodialysis accentuates some of the monocyte abnormalities. This might be the consequence of inflammatory activation through blood-membrane interactions, and biocompatibility issues may play a role. Further, dialysis can clear several but not all retention solutes from the blood of uremic patients. It is thus reasonable to test different dialysis modalities (HDF, high cut-off dialysis) if they influence monocyte dysfunction. All these aspects have been addressed in clinical or experimental studies.

### 4.1. Uremic Retention Solutes and Serum Factors

Uremic plasma strongly influences apoptosis rates of monocytes in cell culture [50]. Several studies showed that uremic plasma nearly doubles apoptosis rates [70,71,72]. This effect seems to be attenuated when the plasma donors are treated by high flux compared to low flux dialysis [71].

Plasma from dialysis patients induces several cytokines and activation markers in monocytes in vitro. It was suggested that the plasma content of advanced oxidated protein products and advanced glycated proteins plays a major role in this monocyte activation. This was supported by the incubation of monocytes with artificially modified human serum albumin that led to enhanced oxidative stress and respiratory burst [73].

Incubation of THP1 monocytic cell line with uremic plasma did not only induce a number of cytokines (IL-6, TNF-α) but modified the surface marker pattern similar to what is noticed in monocytes from patients with CKD [48]. Among these surface markers, there is also a marked induction of ACE expression and reduction of ACE2.

While experiments using plasma do not reveal particular mechanisms of monocyte activation, some effects can be tracked down to well defined retention products. Jankowski and coworkers observed a marked inhibition of inducible nitric oxide (NO) synthetase in monocytes incubated with uremic plasma [74]. They fractionated hemofiltrate and submitted it to gas chromatography and mass spectrometry. The inhibitory effect was shown to be caused by phenylacetic acid. This compound could be confirmed to be a uremic toxin that inhibits monocyte phagocytosis [75].

This is a rare example where uremic plasma effects could be tracked down to a particular chemical substance. Most studies describe effects of uremic serum on the whole, e.g., on expression of microRNA-33a and its target genes adenosine triphosphate-binding cassette transporter A1,G1 (ABCA1, ABCG1) in THP-1 macrophages [76].

A recent study showed that serum from patients with renal failure can induce high expression of ACE on monocytes [48]. The effect seems to be closely related to the induction of high level production of the micro-RNA miR-421 by monocytes. Interestingly, miR-421 expression can be induced by the typical uremic toxins indoxyl sulphate, p-cresol (pCS), and p-cresyl sulphate (IS) [77]. pCS and IS are prominent representatives of protein-bound uremic toxins. While pCS induces the basal level of cellular oxidative stress [78], IS spurs leucocyte-endothelial-interactions and induces monocytic inflammation via p38 MAP kinases (mitogen activated protein kinases)—a central pathway triggering inflammatory cytokines such as IL-1ß and TNF-α [79]. The inflammatory nature of IS was demonstrated by other groups. He and colleagues showed that IS induced inflammation via the retinoic acid-inducible gene/NF-κB pathway [80]. Kim et al. proved that monocytes respond to IS by the aryl hydrocarbon receptor followed by production of TNF-α. TNF-α enhanced the expression of the chemokine ligand CX3CR1L by endothelial cells. This, in turn, attracted activated T-cells which induced endothelial apoptosis [81].

Among the water-soluble toxins (<500 dalton) asymmetric dimethylarginine (ADMA) plays an important role in the progression of kidney and cardiovascular diseases. It is regarded as a key modulator of NO metabolism, contributing to oxidative stress and apoptosis [82]. ADMA mediates adhesion of monocytes to the endothelium by up-regulation of chemokine receptors [83]. Another important representative of uremic toxins <500 dalton is homocysteine. Hyperhomocyteinemia (HHcy) is characteristic feature of renal failure patients and linked to cardiovascular diseases. Recently, Yang et al. impressively demonstrated a relationship between HHcy and CD40-positive monocytes. CD40 is a marker that is highly expressed in antigen-presenting cells and interaction of CD40 with its ligand CD40L activates T-cells. In their article, the authors show that CD40+ monocytes are an inflammatory subset similar to intermediate CD14++CD16+. The CD40/CD40L axis is linked to CVD in CKD. A neutralizing antibody against CD40L prevented the differentiation of monocytes towards this inflammatory CD40+monocyte subtype. By this way, the authors illustrate a potential strategy of blocking an inflammatory pathway in CKD patients with cardiovascular disease (CVD) [17].

### 4.2. The Role of Hemodialysis Treatment on Monocytes Phenotype and Function

Hemodialysis should influence uremic intoxication by removing several solutes in the low to middle molecular weight range. Some studies therefore tested if a hemodialysis session can improve functional parameters in monocytes. However, since clearance of solutes always coincides with blood membrane contact there is the possibility of opposing effects. Contact between blood and dialyzer membranes can induce monocyte activation. Since this activation can be inhibited by treatment with antioxidants, such as superoxide dismutase, it is most likely that oxidative mediators are involved [84].

Carracedo and coworkers studied the role of blood-membrane contacts for the induction of inflammation and apoptosis in monocytes. They incubated THP-1 cells [85] or monocytes [86] in vitro with cuprophane or AN69 dialyzer membranes. This led to different levels of induction of intracellular protein phosphorylation and apoptosis. The activation of caspase-3 is a hallmark of apoptosis. Its activity was raised in cells from patients treated with cellulosic dialyzers. The activation could also be induced by incubating monocytes from healthy individuals in cuprophane mini-dialyzers [87]. Mononuclear cells of patients treated with Cuprophane dialyzer membranes also show a reduced telomer length, which is a characteristic of senescent cells. This feature goes along with increased production of IL-1ß, IL-1RA, and IL-6 [88].

Nowadays the use of biocompatible synthetic membranes is a therapeutic standard in dialysis. When less biocompatible cellulosic membranes were widely used, blood membrane contacts contributed significantly to inflammatory activation of monocytes. Differences between membrane types were described by a number of studies [89,90,91,92,93], among them are also membranes coated with the antioxidant vitamin E [94,95].

Another important feature of dialyzer membranes is their capacity to activate leukocytic adhesion molecules. Adhesion molecules such as CD11b, CD18, and CD62L are a prerequisite for the cells to adhere to the activated vascular endothelium. This is the first step to transendothelial migration. Stavropoulos et al. examined the expression of the adhesion molecules CD11b, CD18, and CD62L before and after a hemodialysis session with either low- or high-flux membranes [96]. While CD62L expression decreased, the other molecules were found at higher expression levels after dialysis. There were no differences between the membrane types.

This study is an example of the difficulties of such experiments: The membranes have different permeability characteristics, however due to their different membrane material, they can also differ in cellular activation signals. Measuring monocytes before and after a dialysis session can be influenced by the dialysis induced sequestration of cells which may heavily change the cell population distribution in peripheral blood. When hemodialysis was introduced as a regular treatment for patients with CKD, profound leukopenia was noted as an immediate complication [97]. Early studies showed that complement activation due to contact of blood with bioincompatible membranes led to granulocyte and monocyte activation. These cell types then adhered to the vascular endothelium, in particular to the capillary endothelium of the lung. Rather few cells may also sequester within the dialyzer module at the membrane [98]. This effect was termed “leukocyte sequestration”, it correlates well with biocompatibility of the dialyzer membrane. Several studies could show that the extent of sequestration is related to the upregulation of adhesion molecules such as P-selectin [99] or CD11b on circulating cells [100,101,102,103].

**Figure 2:**
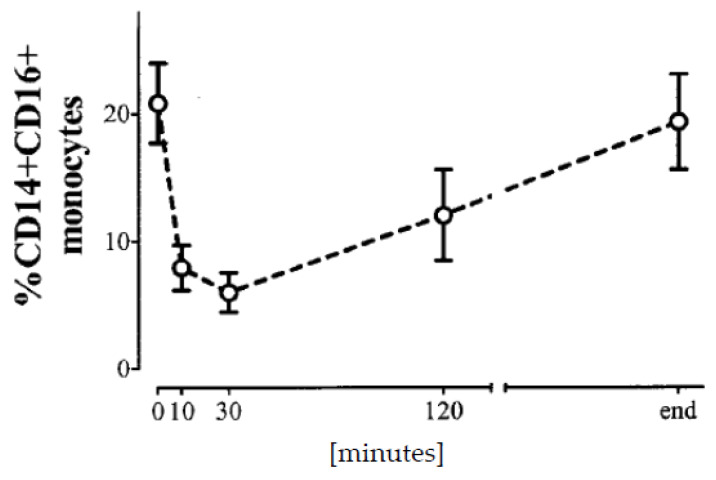
Time course of circulating Mo3 cell numbers during dialysis sessions (Data from: [105]).

Nowadays, granulocyte sequestration no longer occurs since the membranes are much more biocompatible. Nevertheless, there is still some sequestration of monocytes [5], in particular the proinflammatory populations [104]. Obviously, the Mo2 and Mo3 cell populations are much more likely to be removed from circulation during dialysis [64,105] than Mo1 cells. This effect has been suggested as a measure of dialyzer membrane biocompatibility [106]. As a consequence of sequestration, the cells that remain in the circulation during dialysis produce less cytokines compared to cells from blood samples drawn before a dialysis session [107]. The Mo3 cells reach a nadir at about 15–30 min of a dialysis session and return to predialysis numbers until the end of treatment at 4–5 h (Figure 2) [105]. It is not known whether sequestered cells are released from the endothelium or new cells are spilled out of reservoirs such as the spleen. This sequestration effect has to be considered in all studies that follow cellular or cytokine parameters during a dialysis treatment.

These influences can hardly be controlled experimentally. Therefore, interpretation of pre-post studies has to be done carefully. This holds also true for a study that did not find differences in expression of the proto-oncogene bcl-2 in monocytes [108]. Since the expression increased in lymphocytes (thus indicating inflammatory activation) the lack of effect on monocytes might be caused by sequestration of the activated cells from the circulation. A study on the effect of a dialysis session on monocyte HLA-DR and other markers cannot be interpreted at all since at least part of the findings are likely caused by sequestration [5].

Given these difficulties, all study protocols should measure cytokine expression, monocyte population distribution or other markers preferably before a dialysis session. The effects of different membrane characteristics could then be evaluated without influences from sequestration. Studies comparing the use of high cut-off versus high-flux dialysis membranes on the cytokine production profile [109] and the angiotensin-system [110] found less inflammation and a modification of the ACE/ACE2 relation on monocytes towards the normal situation. This might be taken as a hint that the alterations in dialysis patients may be improved by dialysis technology that removes middle molecules (15–45 kD molecular weight).

### 4.3. Further Factors That Influence Monocyte Activation

A plethora of further factors have been described that may contribute to the particular phenotype and function of monocytes in CKD. Among them are potential infectious stimulation by colonized central venous catheters [111], cell free DNA [112], or endotoxin that enters the blood stream through the dialyzer membrane from dialysis fluid [113].

The role of therapeutically applied iron is controversial, activation of monocytes is at least possible [114,115], however, seems to be dose-dependent and avoidable [116]. Uremia leads to several modifications of lipoproteins, among them oxidization. Oxidized LDL is associated with activated monocytes and macrophages [117]. The altered HDL composition in plasma also influences monocyte activation [118].

The molecular effects of vitamin D are a further controversial field in terms of monocyte activation. Vitamin D appears to be able to modulate the immune response by down-regulation of HLA-DR expression [119]. Another study demonstrated that vitamin D exerts anti-inflammatory effects in diabetic nephropathy via TLR/NF-κB signaling pathways [120]. Observations in a small patient number with vitamin D deficiency suggested that repletion might influence monocyte activity as serum levels of inflammatory leucocyte-derived cytokines, such as IL-8, IL-6, and TNF-α, were decreased [121]. Meanwhile, a small 12-week prospective trial showed reduction of IL-6 production via supplementation of vitamin D deficiency with 25OH-vitamin D [122], another placebo-controlled study with the same approach neither showed changes in cytokine production nor monocyte CD14/CD16 population distribution [123].

## 5. Consequences of Monocyte Alterations

### 5.1. Antimicrobial Defense

Multiple phenotypic and functional observations contribute to the explanation of the clinical aspects of monocyte dysfunction in patients with CKD. These clinical consequences are a profound immune defect leading to a high morbidity from infectious diseases, and progressive atherosclerosis. Many aspects of immunologic dysfunction have been discussed above, e.g., the reduced signaling of monocytes during T-lymphocyte activation. This is in part caused by diminished expression of the signaling molecule B7-2 [18] and contributes to impaired vaccination results in these patients against hepatitis B or influenza. Other examples are reduced phagocytosis [46] that might be important in defense against bacterial infection, or monocyte antiviral activity [124].

Circulating monocytes are not a professional antimicrobial cell type. However, after tissue invasion through the endothelium, they differentiate into dendritic cells (DC). These cells are highly important for activation and regulation of the antigen-specific immune defense. A few studies have tested the capacity of monocytes from patients with renal failure to differentiate into DC in vitro. They found that these cells differentiate more quickly to DC [125], however they also suggest that this differentiation may not entirely follow the functional path of cells from healthy individuals. Choi et al. provided information about the stimulatory capacity of mature mo-derived dendritic cells (mo-DCs). They demonstrated that an inflammatory cocktail consisting of IL-1ß, Il-6, TNF-α and prostaglandin E2 led to significant higher IL-6 production in HD patients compared to healthy controls [126]. Further on, incubation with uremic sera decreased endocytosis and increased IL-12p70 production in mo-DC from healthy donors. Mo-DC isolated from HD patients and incubated with healthy sera showed reduced endocytosis and produced higher amounts of IL-12p70 than mo-DC from healthy donors [127]. Regarding mo-DC, the impairment of these cells have profound clinical consequences. This abnormality is related to the impaired Hepatitis B vaccination, probably because of an impairment of mo-DCs to stimulate antigen-specific T cells [128]. This is very plausible since DCs are involved in tissue antigen presentation after intramuscular injection of vaccine.

### 5.2. Monocyte Activation Contributes to Atherosclerosis

Monocytes and macrophages strongly contribute to the pathogenesis of atherosclerosis. These cells invade the vessel wall at sites of endothelial lesions and participate in the formation of foam cells, the deposition of lipids in the intima, and the growth of the atheromatous plaque [129]. Macrophages within vascular plaques express inflammatory markers [130]. These observations prompted the study of a relationship between the activation of circulating monocytes in the blood of dialysis patients and their risk of atherosclerotic vascular disease. Investigators found many hints that such relation exists. Studies in dialysis patients described correlations between monocyte expression of IL-1 and IL-1RA and cardiovascular events [131]. The relative frequency of CD14^++^/16^+^ Mo2 cells relates to this morbidity in different cohorts with CKD including HD, PD and CKD patients stages 1–5 [52,132,133]. A high expression of ACE on monocytes appears to be associated with cardiovascular disease in HD and PD patients [25,61,134]. In addition, the intensity of monocyte sequestration during a dialysis session as a surrogate for the activation level of this cell type is predictive for cardiovascular events as well [135]. Nevertheless, negative studies must be noted as well. The number of TLR-4 positive monocytes in the circulation was not predictive for cardiovascular mortality in CKD patients stage 5 [136].

While these associations do not establish causality, some findings at least suggest that there could be direct an involvement of the cell type in the pathogenesis of atherosclerosis. Monocytes from dialysis patients highly express the scavenger receptor CD36 [137] or at least show high transcription for this molecule [138]. The scavenger receptor is important for the uptake of LDL-cholesterol into monocytes and macrophages which leads to foam cell formation. Monocytes from renal failure patients differentiate into highly activated, inflammatory and cytokine producing foam cells in vitro [139]. Monocytes are also more prone to adhere to endothelium and to transmigrate in chamber experiments [140]. These findings are closely associated with ACE expression on this cell type [45]. Finally, markers of monocyte activation could also be shown in the vessel wall itself in dialysis patients who had vascular surgery for atherosclerotic artery disease [141]. Vessel invading monocytes were positive for the markers anti-CD14 and ant-CD68. Further on, monocytes expressed the post-translationally modified acetylated form of the Y-box binding protein-1 (YB-1). This protein is known to regulate the transcription of the C-C chemokine 5 (CCL5/Rantes) gene which serves as a chemoattractant during inflammation and atherosclerosis.

While monocytes may contribute to ischemic vascular disease by promoting atherosclerotic plaque growth, the cells can also enhance platelet activation and support thrombotic vessel occlusion. Platelets and monocytes seem to form aggregates in the circulation and the activation of platelets parallels that of monocytes. The formation of these aggregates was higher in HD compared to PD patients [142,143]. In one study the extent of such activation correlated with the cardiovascular event risk in hemodialysis patients [142]. These aggregates between monocytes and platelet are formed during dialysis while platelet P-selectin expression increases in HD patients [144]. However, not all groups could confirm these findings [145].

## 6. Conclusions and Perspectives

Regarding monocytes, impairment and activation are two sides of the same coin—uremia. A decrease of phagocytic capabilities, impairment of antigen presentation function on the one side and elevation of inflammatory markers and specialized monocyte subsets on the other side characterize the monocyte momentum in CKD. Functionally, the cells are equipped with a machinery for production of inflammatory cytokines and adhesive/migratory receptors, thus being able to enhance and transfer inflammation to sites of vascular damage. This can result in initiation and progression of cardiovascular diseases, most prominently atherosclerosis. Since dialysis contributed to monocyte alterations, the improvement of biocompatibility of the treatment has been a valid approach in the past. However, CKD itself, including the retention of middle molecules and uremic toxins, remains a major pathogenetic cause of inflammation and impaired immune defense. Several studies address the potential of anti-inflammatory intervention to reduce atherosclerotic cardiovascular disease [146]. Such studies should be accompanied by the monitoring of monocyte function in the future.

## 7. Methods

Since the available literature is highly heterogenic in choice of methods and reporting, the form of a narrative review was chosen. Literature was identified using the following PubMed query:

(“Macrophages”[Mesh] OR “Monocytes”[Mesh]) AND (“Kidney Failure, Chronic”[Mesh] OR “Renal Dialysis”[Mesh])

The results from this query (*n* = 948) were filtered using the following filters: with abstract, English language, Humans, time range 01.01.1994–31.12.2019. The resulting 466 entries were read and evaluated. Studies on the pathogenesis of glomerular diseases, infectious complications (mainly peritonitis in peritoneal dialysis), kidney transplantation, renal osteodystrophy, and studies not mentioning monocytes were excluded. We further excluded case reports and review publications not reporting primary data.

## Figures and Tables

**Table 1:** Comparison of monocyte surface expression density (mean fluorescence intensity) in pre-dialysis samples compared to healthy control persons. Studies that reported % positive cells only without giving expression density are marked with an §. One study measured transcriptional activity for the given protein instead of surface expression by flow cytometry, this study is marked with an #. ↓, down-regulation, ↑, up-regulation, →, unchanged.

Surface Marker	Function	Comparison to Healthy Control	Quote	Year of Publication
CD11b	Integrin, adhesion molecule	↓ →	[9] [10,11]	2000 2000, 2010
CD14	Endotoxin receptor	↑ ↓	[12] [13] # [10]	2002 2015 2010
CD16	Immunoglobulin Fc receptor γIII	→	[13]	2015
CD31	PECAM-1 endothelial adhesion	→	[14]	2001
CD36	“scavenger” receptor of oxidized lipoproteins	↑	[15,16]	2005
CD40	Receptor for co-stimulating signals of B-cells, promotes cytokine production in macrophages	↑	[17]	2016
CD68	Gp110, function?	↑	[16]	2005
CD86	B7-2, co-stimulation of T-cells	↓	[18]	2001
CD95	Fas, apoptosis induction	↑	[16]	2005
HLA-DR	Class II tissue antigen, antigen presentation	→ ↑	[18] [10,19]	2001 2008, 2010
MAC-1	CD11b/CD18 dimer, adhesion, complement receptor	↑	[14]	2001
TLR-2	Toll-like receptor, recognition of bacteria etc.	→ ↑	[20] [21,22]	2007 2010, 2011
TLR-4	Toll-like receptor, LPS-receptor	↓ → ↑	[20] [22] [21]	2007 2011 2010
TNF-R2	Receptor for TNF-α	↑	[4,23]	2001, 2005
CX3CR1	Fractalkine receptor, adhesion molecule	↑	[13] #	2015
CCR2	C-C chemokine receptor 2	↑	[24] §	2009
ACE	Angiotensin converting enzyme	↑	[25]	2006
AChR	Receptor for Acetylcholine	↑	[26]	2016

**Table 2:** Cytokine production into the supernatant of cultured monocytes from the blood of hemodialysis patients compared to those from healthy controls. IL = interleukin, TNF = tumor necrosis factor, TGF = transforming growth factor, PTX = pentraxin; * = single cell intracellular measurement. ↓, down-regulation, ↑, up-regulation, →, unchanged.

Cytokine	Function	Unstimulated	Stimulated by LPS	Quote
IL-1ß	Proinflammatory	↑	↓	[27]
IL-6	Proinflammatory	→ * ↑	→ * →	[28] [27,29,30]
TNF-α	Proinflammatory	↑	→ → * ↑	[27] [31] [30]
TGF-ß	Anti-proliferative, profibrotic	↑		[29]
IL-10	Anti-inflammatory	→ *	→	[28]
PTX-3	Pattern recognition, antibacterial defense	↑	→	[32]

**Table 3:** Protein content or transcriptional activation for regulatory or anti-inflammatory/antioxidative systems in monocytes from patients with CKD compared to healthy individuals.

System	Function	Comparison to Healthy Controls	Quote
SOD1	Superoxide dismutase 1, antioxidative	Protein content low, transcription rate high	[39]
SOD2	Superoxide dismutase 2, antioxidative	Reduced protein content CKD3/4, normal CKD5D; enhanced transcriptional activation all CKD	[40]
Rhodanese	Regulation of mitochondrial reactive oxygen species production	Protein content and transcription low	[41]
Hsp72	Heat shock protein 72, protein folding and degradation, cellular damage protection	Protein content and transcription low	[42]
SOCS3	Suppressor of cytokine signaling, modulates intracellular signaling after cytokine-receptor interaction	Enhanced transcription	[43]
Lp-PLA2	Lipoprotein-associated Phospholipase A2, platelet activation, pro-atherogenic	Enhanced transcription	[44]
ACE ACE2 AT1-R AT2-R	Angiotensin converting enzyme Angiotensin converting enzyme type 2 Angiotensin II receptor Type 1 Angiotensin II receptor Type 2	Enhanced transcription Reduced transcription Enhanced transcription Normal transcription	[45]
